# A survey on patients' characteristics, perception of family support and diabetes self‐management among type 2 diabetes patients in South‐West Nigeria

**DOI:** 10.1002/nop2.236

**Published:** 2019-01-07

**Authors:** Lucia Y. Ojewale, Abimbola O. Oluwatosin, Adesoji A. Fasanmade, Olatunde Odusan

**Affiliations:** ^1^ Department of Nursing, College of Medicine University of Ibadan Ibadan Nigeria; ^2^ Department of Medicine, College of Medicine University of Ibadan Ibadan Nigeria; ^3^ Department of Medicine, College of Medicine Olabisi Onabanjo University Teaching Hospital Sagamu Nigeria

**Keywords:** diabetes self‐management, family support, south‐west Nigeria, type 2 diabetes

## Abstract

**Aim:**

To determine the association between patients' characteristics, perception of family support and diabetes self‐management (DSM) behaviours among type 2 diabetes patients.

**Design:**

A descriptive cross‐sectional design was used and data were collected between July–September 2016. The study is part of a larger quasi‐experimental study.

**Methods:**

One hundred and ninety‐seven diabetes mellitus (DM) patients from two teaching hospitals in south‐west Nigeria participated. Questionnaire was used in collecting information on sociodemographic, clinical data, DSM and perception of family support.

**Results:**

Most (71.6%) of the participants were females and 35% were on insulin therapy. Mean age was 60.7 (*SD*: 11.3) years and 11.7% had had DM for over 20 years. Overall, DSM was positively influenced by previous diabetes education and duration of diabetes. Perception of family support was also positively associated with and influenced DSM.

## 1 INTRODUCTION

Diabetes mellitus (DM) is a chronic, lifelong condition which requires that patients make daily decisions regarding the management. These decisions include appropriate dietary intake, physical activity and adherence to medications, oftentimes with minimal input from a healthcare professional (Jarvis, Skinner, Carey, & Davies, 2010). The disease is on the increase in Nigeria and complications are widely reported (Chinenye et al., 2012). Several factors including patients' personal characteristics and social environment are linked with diabetes self‐management (DSM) among people living with diabetes (PLWD).

Individuals with DM like others live with and interact with their family members. This interaction can have an impact on the way the disease is managed (Adejoh, 2012). In many healthcare facilities in Nigeria, individuals with diabetes are provided with diabetes self‐management education (DSME) by nurses at the point of diagnosis and during follow‐up visits. This education is often structured so that all the important aspects of the condition are well explained to the patient. The influence of family members on diabetes management and glycaemic control (positive or negative) has been documented by some authors (García‐Huidobro, Bittner, Brahm, & Puschel, 2010; Mayberry & Osborn, 2012; Tang, Brown, Funnell, & Anderson, 2008). Yet, family members often play passive role during patient education, if at all they attend the clinic with the patient.

Evidence supporting the important role of the family in DSM in south‐west Nigeria is limited, and data on the extent to which patients' personal factor affects self‐management are also scarce. Examining these aspects of diabetes care could help to better focus diabetes management towards increasing adherence and reducing complications of the disease. Specifically, it could be a spur for nurses and other relevant stakeholders in the hospital to provide a more structured education for the family members of individuals with diabetes.

## 2 BACKGROUND

Globally, 425 million adults (8.8%) between ages 20–79 years are living with diabetes mellitus (DM) which cause an annual death of about 4.0 million worldwide (International Diabetes Federation [IDF], 2017). Out of the total number of adults with diabetes recorded in 2017 by IDF, Nigeria had the highest prevalence in the West African region—1.7 million as are the disease complications. A high prevalence of peripheral neuropathy, retinopathy and cataracts, among others, was found among DM patients in a multi‐site study involving seven tertiary hospitals in the country (Chinenye et al., 2012).

If well managed, PLWD can live healthy and useful life like others. Effective self‐management includes adherence to diet, medications, exercise and self‐monitoring of blood glucose (Schmitt et al., 2013). Self‐management has been defined as “the ability of a person in conjunction with family, community and health professionals to manage symptoms, treatments, lifestyle changes and psychosocial, cultural and spiritual consequences of a health condition” (Richard & Shea, 2011). For instance, self‐glucose monitoring has been shown to assist patients in modifying food, exercise and medications (Musenge, Michelo, Mudenda, & Manankov, 2016), as knowledge about illness and status has been shown to be associated with change in perception of illness, which is further linked with lifestyle modifications and blood glucose control (Malanda et al., 2012).

Similarly, patients who adhere to medication have better glycaemic control (Pascal, Ofoedu, Uchenna, Nkwa, & Uchamma, 2012). Furthermore, exercise is an important aspect of diabetes care because it is associated with better glycaemic control, prevention of cardiovascular risks and a sense of general well‐being (American Diabetes Association [ADA], 2013). ADA further stated that diet modification is central to DSM.

Various personal (sociodemographic and clinical) and psychosocial factors affect self‐management among diabetes patients. For instance, high educational attainment was associated with increased physical activities, healthy diet and better foot care (Mogre, Abanga, Tzelepis, Johnson, & Paul, 2017). Okolie, Ehiemere, Ezenduka, and Ogbu (2010) also found a higher dietary adherence among males compared with females, married individuals compared with those unmarried, those unemployed versus employed patients, diabetes patients aged 18–50 years in contrast with those over 50 years. Okolie et al. (2010) reported that individuals with primary school or no education adhered better than those with secondary and tertiary education. This is contrary to the report of Mogre et al. (2017). Abubakari, Cousins, Thomas, Sharma, and Naderali (2016) further documented the association between diabetes duration and self‐management although Huang, Zhao, Li, and Jiang (2014) found the opposite among Chinese participants. Moreover, there is strong support in literature that previous exposure to DSME is associated with DSM (ADA, 2012; Davies et al., 2008; Odili & Eke, 2010).

In addition, the social environment where the patient interacts with others plays a major role in DSM as the patient can influence and be influenced by others (Rintala, Jaatinen, Paavilainen, & Astedt‐Kurki, 2013). The effects of family members' support on self‐management of diabetes patients can be either positive or negative. Reduction in glycosylated haemoglobin (HbA1c) signifying optimum glycaemic control, better knowledge of diabetes and improved quality of life are some of the positive influences family members have had on diabetes patients (García‐Huidobro et al., 2010; Tang et al., 2008). On the other hand, destructive and/or non‐supportive behaviour among family members, whereby patients feel sabotaged by family members or were offered help that reduced their self‐efficacy have been reported (Harris, 2006; Mayberry & Osborn, 2012).

Some studies have also described the advantages of social support in diabetes care and education. Beverly, and Wray (2010) reported that family members of people with diabetes assisted them with exercise. Stephens, Rook, Franks, Khan, and Iida (2010) and Watanabe et al. (2010) also documented the assistance provided by family members in ensuring adherence to diabetes diet. In addition, García‐Huidobro et al. (2010) reported a reduction in diabetes patients' A1C as a result of family support. High level of family/friend social support was associated with higher DSM, though not associated with A1C in the study by Vaccaro, Exebio, Zarini, and Huffman (2014).

Studies on the association between family support and DSM are limited in Nigeria, particularly in the south‐west region. The few studies published include that by Okolie et al. (2010) who reported that lack of spousal support limited self‐care among diabetes patients in the Eastern part of Nigeria. On the other hand, another study by Adejoh (2012), which took place in north central Nigeria, family support had a negative influence on diabetes care; although in the qualitative aspect of the study, the patients reported mixed feelings about family support. One study in south‐west Nigeria examined and reported that fasting blood glucose was associated with perception of family support (Adetunji, Ladipo, Irabor, & Adeleye, 2007). However, the factor that precedes effective blood glucose level, that is DSM, has not been examined in association with perception of family support.

In addition, even though some of the aforementioned authors—Okolie et al. (2010), Adejoh (2012)—have reported self‐management among diabetes patients in Nigeria, none has used the DSM scale which is a standardized instrument that fully encompasses the four main domains of diabetes management. The scale, developed by Schmitt et al. (2013), has been shown to be effective in predicting the level of self‐care activities which correspond to good glycaemic control. Therefore, the research questions are as follows:

- What is the association between selected patient characteristics and DSM among type 2 diabetes patients in south‐west Nigeria?
- What is the association between perception of family support and DSM among type 2 diabetes patients in Nigeria?

## 3 THE STUDY

### 3.1 Design

The study was a cross‐sectional multi‐centre survey, which is part of a larger quasi‐experimental study. Data were collected using standardized/pretested, close‐ended questionnaire consisting of three main sections, viz sociodemographic and clinical data, DSM and perception of family support.

### 3.2 Data collection

The study took place at two teaching hospitals in south‐west Nigeria—University College Hospital (UCH), Ibadan, Oyo State, and Olabisi Onabanjo University Teaching Hospital (OOUTH), Sagamu, Ogun State. The UCH is the only Federal Government tertiary health institution in Ibadan, Oyo State, Nigeria. It mainly serves as a referral centre for other healthcare facilities within and outside Oyo State. The diabetes clinic holds twice a week—Mondays and Fridays—and an average number of 60 patients attend the clinic of the hospital on a weekly basis. OOUTH also acts as a referral centre for healthcare facilities within and outside Ogun State. An average of 40 patients attend the clinic on a weekly basis—Tuesdays.

The sample size for this study is that calculated for the larger quasi‐experimental study (yet to be reported). This was based on a statistical power of 90% with the goal of demonstrating an expected effect of 25% decrease in prevalence of suboptimal glycosylated haemoglobin (HbA1c) level.

The study sample consisted of 197 type 2 DM patients aged 18 years and above attending follow‐up clinics at the two diabetes clinics between July–September 2016. Patients with cognitive impairment and those who were not living with/accompanied by any family member were excluded. The questionnaire was in English with translation into native (Yoruba) language. It was self‐administered by literate patients and administered by four trained research assistants to unlettered patients.

### 3.3 The questionnaire

The instrument for data collection was a questionnaire which comprised of three parts. The first part was on sociodemographic and clinical‐related information. These included age, duration of diabetes, average monthly income, highest level of education, previous exposure to diabetes education, ownership of a glucometer and whether or not on insulin injection, among others.

The second part focused on questions on DSM. This was assessed using the 16‐item Diabetes Self‐Management Questionnaire (DSMQ) developed by Schmitt et al. (2013). The Cronbach's alpha for the instrument was 0.95. The answers to statements on diabetes management were on a 4‐point Likert scale as follows: “Does not apply to me,” “applies to me some degree,” “applies to me to a considerable degree” and “applies to me very much,” with scores ranging from “1–4,” respectively. Where necessary, negatively worded items were reversed. The highest obtainable score for this section was 64, while the lowest was 16.

The final part of the questionnaire was on “Perception of social support from family.” Questions were elicited using the perceived social support, family scale. Perceived family support is the degree to which one perceives how his or her needs for support are fulfilled by family (Afolabi, Abioye‐Kuteyi, Fatoye, Bello, & Adewuya, 2007). The scale was originally developed and validated by Procidiano and Keller (1983). It is a 20‐item questionnaire with a Cronbach's alpha of 0.95 after being modified and adapted. Although the options on the original scale were “Yes” or “No,” the questionnaire was adapted so that statements were scored on a 4‐point Likert scale ranging from “strongly agree” (1) – “strongly disagree” (4). The highest obtainable score was “80,” while the lowest was “20.”

### 3.4 Ethical consideration

Ethical permission was obtained from the UI/UCH ethics committee and OOUTH ethical Review Boards. Official letter of introduction and permission to collect data was obtained from the Head of Nursing Department, University of Ibadan, and presented at the two hospitals. Informed consent was obtained from the patients after the nature of the study had been explained to them. Four research assistants were trained to assist with data collection. To reduce waiting time at the clinic, the researcher and the research assistants arrived early at the clinic, before consultation with endocrinologist started or before it got to the turn of patients who were eligible to participate in the programme. In addition, even though the two clinics receive quite a high number of diabetes patients on each clinic day, only an average of 12 patients were recruited on each day of data collection as majority of the patients did not meet the inclusion criteria. This further prevented patients from having to wait after their normal clinic routine because of the data collection.

### 3.5 Data analysis

Questionnaire was checked for completion and errors on a daily basis after which data were entered into the IBM—SPSS (Statistical Package for the Social Sciences), version 22 computer software for analysis. Categorical variables, such as gender and educational status, among others were summarized using frequencies and percentages. The statistical means of other variables including age, duration of diabetes and average monthly income were first determined. The variables were then categorized using frequencies and percentages. Perception of family support was categorized into “Good” if score was above or equal to the mean and “Poor” if score was below the mean. The categories were presented using bar chart. Responses to the 16 items on the DSM Scale were summarized using percentages and the result presented using bar charts. The mean of the entire group was determined as well. Based on scores below and above the mean, participants' DSM was categorized into “Poor” and “Good” respectively.

Association of sociodemographic and clinical‐related data with DSM was determined using chi‐square. Independent *t* test was used in determining the association between DSM and perception of family support with *p* significant at <0.05.

## 4 RESULTS

The sociodemographic and clinical variables of the 197 type 2 diabetes patients who took part in the study are presented in Table 1. Majority of the study participants were females (71.6%), educational attainment was up to secondary school level only, in most (67%) of the participants, while only 21% of them earned income that exceeded 50,000 naira ($150) monthly. The largest proportion (55.3%) were 60 years and above.

**Table 1:** Sociodemographic and clinical characteristics of the participants (*N* = 197)

Variable	Category	*N*	%
Gender	Male	56	28.4
	Female	141	71.6
Age group (years), mean: 60.7 (±11.3)	≤40	11	5.6
	41–59	77	39.1
	≥60	109	55.3
Highest level of education	Tertiary	65	33.0
	Secondary and below	132	67.0
Monthly income (in naira)	<50,000	155	78.7
	≥50,000	42	21.3
Marital status	Married	142	72.1
	Not married	55	27.9
Diabetes duration (years)	<20	173	87.8
	≥20	23	11.7
Insulin use	Yes	69	35.0
	No	128	65.0
Ownership of a glucometer	Yes	156	79.2
	No	41	20.8
Previous DM education	Yes	161	81.7
	No	38	18.3

Furthermore, 35% of the participants were on insulin therapy, while 81.7% had been exposed to diabetes education and many (79.2%) owned a glucometer. Whereas only 11.7% of them had been diagnosed and receiving treatment for 20 years and above, diabetes duration in 87.8% of the study population was <20 years.

**Figure 1:**
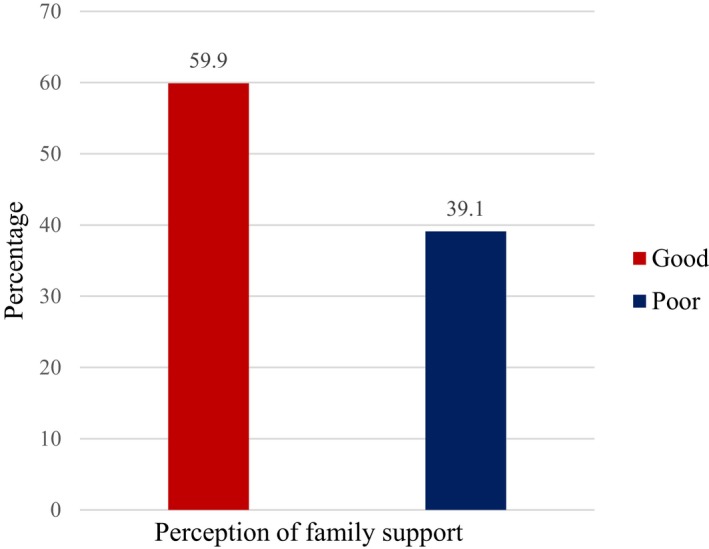
Perception of family support categories

**Figure 2:**
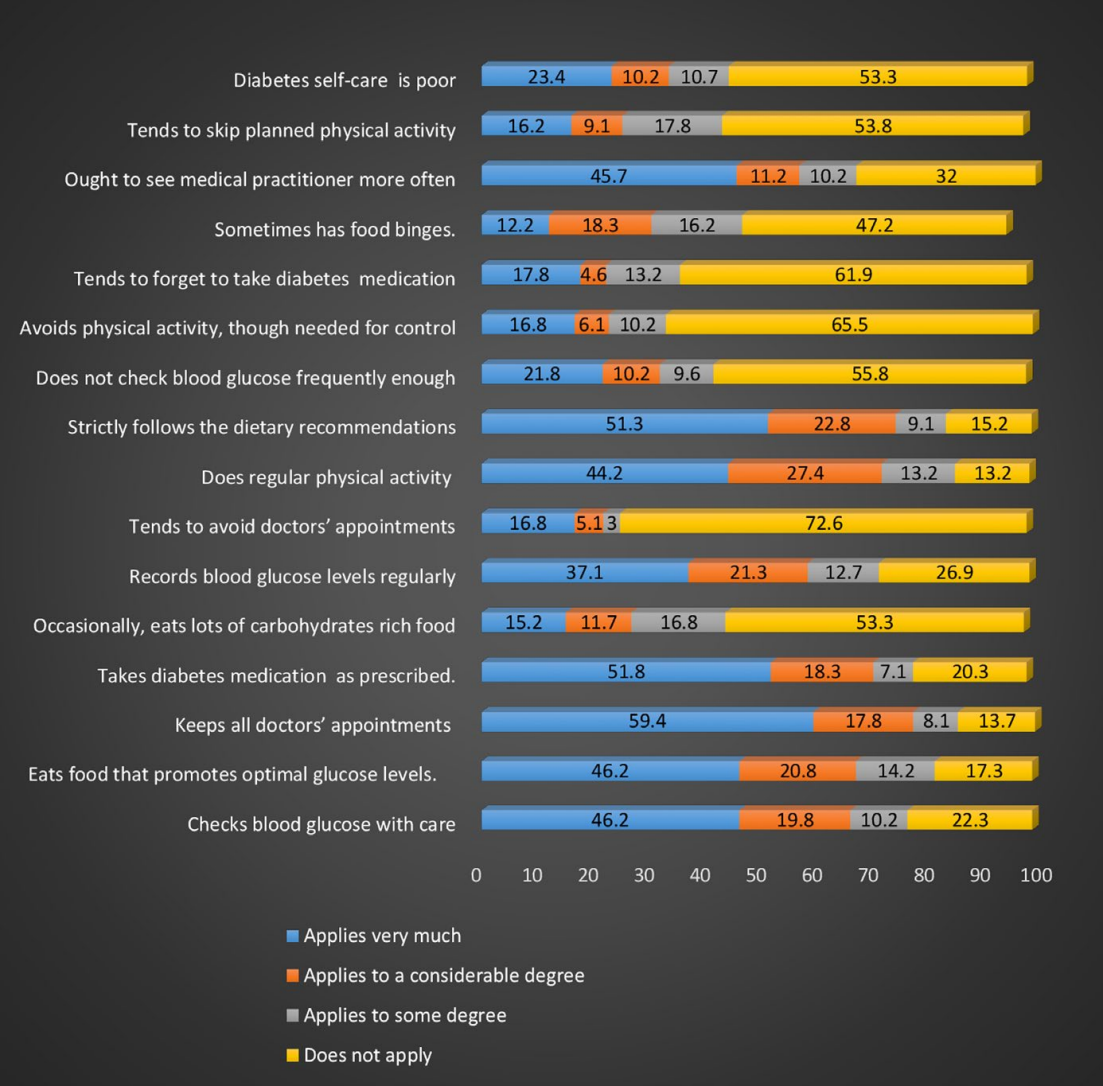
Domains of diabetes self‐management practices

**Figure 3:**
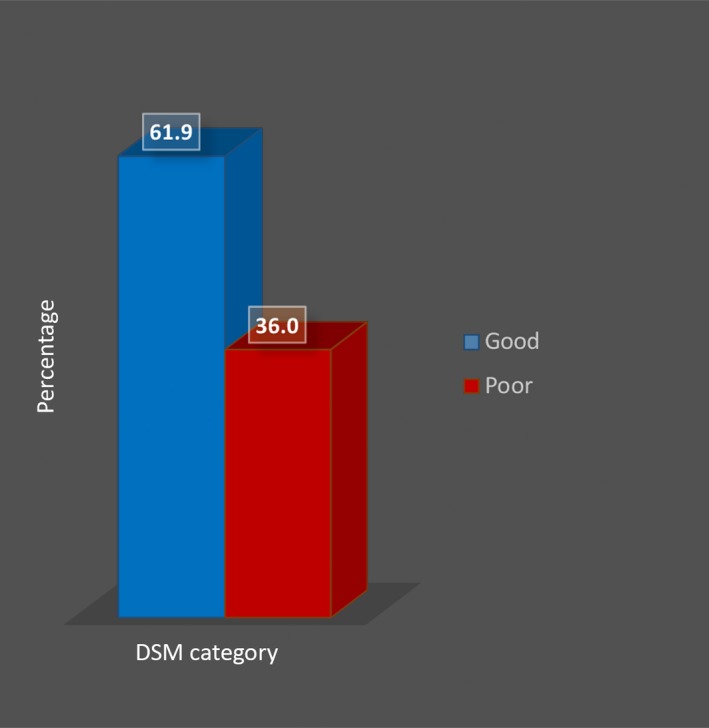
Diabetes self‐management (DSM) categories

A sizable number of the diabetes patients (60%) had a good perception of family support as shown in Figure 1. Result of the domains of DSM practices of the patients shows that 23.4% rated their DSM as being poor (Figure 2, domain 1). In the same figure, items regarding specific DSM domains including adherence to diabetes diet, physical activity, self‐blood glucose monitoring, medication adherence and use of health care are assessed with both positively and negatively worded statements. The responses show similarity in the percentages. The DSM of the patients is further categorized into good and poor and presented in Figure 3 where majority (61.9%) had a good DSM.

The association between sociodemographic as well as diabetes management parameters and DSM is shown in Table 2. The duration of diabetes and exposure to previous diabetes education were associated with DSM (*p* < 0.05). On the other hand, age, gender, marital status, educational attainment, income, use of insulin and ownership of a glucometer were not significantly associated with DSM (*p* > 0.05). Also, as illustrated using Table 3, the DSM of patients with good and poor perception of family support shows a significant difference (*p* < 0.01).

**Table 2:** Association between sociodemographic, clinical variables and diabetes self‐management (DSM)

Variable	Category	DSM		*p* Value
		Good	Poor	
		*f* (%)	*f* (%)	
Gender	Male	32 (26.2)	23 (32.4)	0.41
	Female	90 (73.8)	48 (67.6)	
Age group (years), mean: 60.7 ± 11.3	≤40	5 (4.1)	6 (8.5)	0.27
	41–59	45 (36.9)	30 (42.3)	
	≥60	72 (59.0)	35 (49.3)	
Marital status	Married	91 (74.6)	23 (32.4)	0.32
	Not married	31 (25.4)	48 (67.6)	
Highest level of education	Tertiary and above	38 (31.1)	26 (36.6)	0.53
	Secondary and below	85 (68.9)	45 (63.4)	
Income (naira)	<50,000	91 (74.6)	60 (84.5)	0.15
	≥50,000	31(25.4)	11(15.5)	
Diabetes duration (years), mean: 9.0 (7.7)	<19	102 (84.3)	67 (94.4)	0.04*
	≥20	19 (15.7)	4 (5.6)	
Insulin use	Yes	44 (36.1)	23 (32.4)	0.64
	No	78 (63.9)	48 (67.6)	
Ownership of a glucometer	Yes	93 (76.2)	59 (83.1)	0.28
	No	29 (23.8)	12 (16.9)	
Previous DM education	Yes	105(86.1)	52 (73.2)	0.035*
	No	17 (13.9)	19 (26.8)	

* Statistically significant.

**Table 3:** Independent *t* test for family support and diabetes self‐management (DSM)

Perception of family support	DSM, mean (*SD*)	Mean difference	*p* Value
Good	52.0 (6.1)	2.61	0.007
Poor	49.4 (6.9)		

## 5 DISCUSSIONS

This study reports the association between perception of family support and DSM among 197 type 2 diabetes patients who were recruited from two teaching hospitals in south‐west Nigeria. The survey is part of a bigger study.

Longer duration of diabetes was associated with DSM similar to the findings of Abubakari et al. (2016) but contrary to the findings of Huang et al. (2014). However, the association between previous diabetes education and DSM agrees with the findings of several other authors (ADA, 2012; Davies et al., 2008; Odili et al., 2010; Steinsbekk et al., 2012).

Contrary to Okolie et al. (2010) and Mogre et al. (2017), in this study, there was no association between gender, educational attainment and DSM/adherence. This may be due to the fact that the authors made use of a different scale where different aspects of self‐management—blood glucose monitoring, foot care, diet adherence and adherence to exercise —were individually compared with the sociodemographic parameters, whereas in this study a sum scale encompassing the various aspects of self‐management was used. Similarly, DSM was not associated with older age as opposed to the findings of Abubakari et al. (2016).

Most participants had a high level of perceived social support from family. This may be an evidence of the close‐knit nature of the Nigerian family system (Eboiyehi, 2015). A major question in this study borders on determining the association between perception of family support and DSM. Findings from this study showed a significant association between perception of family support and DSM, supported by a study among Chinese patients despite the fact that the authors used different scales to determine the DSM and perception of family support (Huang et al., 2014). Likewise, this study finding regarding association between DSM and perception of family support is supported by several other authors (Beverly, Penrod, & Wray, 2007; García‐Huidobro et al., 2010; Stephens et al., 2010; Vaccaro et al., 2014; Watanabe et al., 2010), but contrary to that of Adejoh (2012) and Mayberry and Osborn (2012).

The mean DSM score of patients was high with 61.9% of participants having a good self‐management, of which self‐reported DSM comprised diet adherence, exercise, glucose monitoring, medication adherence and follow‐up/use of healthcare facilities. Contrary to this report, only about 16% of diabetes patients in Germany were reported to have a high level of self‐management behaviour while the rest had a low level (Laxy et al., 2014). However, the instrument for assessing the DSM was different from the one used in this study. Whereas we used the DSMQ developed by Schmitt et al. (2013), the authors made use of self‐management behaviour index developed by Arnold‐Wörner, Holle, Rathmann, and Mielck (2008). Gao et al. (2013) found an average level of self‐management among Chinese PLWD using the Summary of Diabetes Self‐Care Activities (SDSCA) questionnaire. However, the SDSCA questionnaire had earlier been criticized for its inability to relate any of its scale with HbA1c (Primožič, Tavčar, Avbelj, Dernovšek, & Oblak, 2012).

## 6 CONCLUSION

Our study findings show that family support is positively associated with DSM. This underscores the need to better involve family members in a structured and formal education to reinforce patient education. Previous exposure to diabetes education was also significantly associated with DSM lending credence to the widespread belief of the importance of educating PLWD on the disease condition.

## 7 RECOMMENDATIONS FOR NURSING PRACTICE

Since diabetes patients feel supported by their family members, this positive relationship can be used as a tool for enhancing DM patients' self‐management of diabetes, through the education of family members. Specifically, family members can be taught to offer concrete support such as assisting with checking blood glucose level using a glucometer, administering insulin injection, among others. Concretizing the support is particularly important since this was not captured in the data collection instrument as the focus was on determining “perception of support.”

In addition, the family members who accompany DM patients to the hospital and receive a well‐structured diabetes education can learn to adopt a healthy lifestyle towards preventing the disease since it is hereditary.

Finally, patients who had been diagnosed of having diabetes for many years, for instance over 10 years, can act as effective peer educator. Although this practice already takes place in some countries, it has not really taken shape in many others including Nigeria.

## 8 LIMITATION

The study is limited by the relatively small sample size.

## PATIENT CONSENT

Informed consent was obtained from participants prior to data collection.

## CONFLICT OF INTEREST

No conflict of interest has been declared by the authors.

## AUTHOR CONTRIBUTIONS

LYO: Concept and design contribution, data collection and analysis, and drafting and revision of the manuscript. AO: Concept, design, analysis contribution and revision of the manuscript thoroughly. AF: Design contribution, data collection and revision of the manuscript critically. OO: Data collection and revision of the manuscript critically as well.
